# An open multi-center MEG-EEG dataset for studying conscious visual perception

**DOI:** 10.1038/s41597-026-07350-9

**Published:** 2026-05-29

**Authors:** Ling Liu, Oscar Ferrante, Tara Ghafari, Dorottya Hetenyi, Shujun Yang, Rony Hirschhorn, Urszula Gorska-Klimowska, Praveen Sripad, Fatemeh Taheriyan, Tanya Brown, Diptyajit Das, Kyle Kahraman, Niccolò Bonacchi, Michael Pitts, Liad Mudrik, Ole Jensen, Huan Luo, Lucia Melloni

**Affiliations:** 1 Cognitive Science and Allied Health School, Beijing Language and Culture University, Beijing, 100875 China; 2 Speech and Hearing Impairment and Brain Computer Interface LAB, Beijing Language and Culture University, Beijing, 100875 China; 3 School of Psychological and Cognitive Sciences, Peking University, Beijing, 100871 China; 4 Centre for Human Brain Health, School of Psychology, University of Birmingham, Birmingham, B15 2TT UK; 5 School of Psychology, University of Surrey, Guildford, GU2 7XH United Kingdom; 6 Department of Experimental Psychology, University of Oxford, Oxford, OX2 6GG UK; 7 Oxford Centre for Human Brain Activity (OHBA), Oxford Centre for Integrative Neuroimaging (OxCIN), Department of Psychiatry, University of Oxford, Oxford, OX3 7JX UK; 8 Department of Imaging Neuroscience, UCL Queen Square Institute of Neurology, University College London, WC1N 3AR London, UK; 9 Department of Psychology, University of Amsterdam, Amsterdam, Netherlands; 10 Sagol School of Neuroscience, Tel Aviv University, Tel Aviv, 6997801 Israel; 11 Department of Neurology, University of Wisconsin-Madison, Madison, WI 53705 USA; 12 Neural Circuits, Consciousness and Cognition Research Group, Max Planck Institute for Empirical Aesthetics, Frankfurt am Main, 60322 Germany; 13 Interdisciplinary Center for Neuroscience Frankfurt, Heinrich-Hoffmann-Straße 7, 60528 Frankfurt am Main, Germany; 14 William James Center for Research, ISPA - Instituto Universitario, Lisbon, 1149-041 Portugal; 15 Champalimaud Research, Lisbon, 1400-038 Portugal; 16 Psychology Department, Reed College, Portland, OR 97202 USA; 17 School of Psychological Sciences, Tel Aviv University, Tel Aviv, 69978 Israel; 18 Program for Brain, Mind, and Consciousness, Canadian Institute for Advanced Research, Toronto, Ontario Canada; 19 Department of Neurology, New York University Grossman School of Medicine, New York, NY 10016 USA; 20 Predictive Brain Department, Research Center One Health Ruhr, University Research Alliance, Faculty of Psychology, Ruhr University Bochum, Bochum, 44801 Germany

**Keywords:** Consciousness, Object vision

## Abstract

Here, we present a large-scale, multi-center dataset of combined magnetoencephalographic (MEG) and electroencephalographic (EEG) recordings, along with eye-tracking data and high-resolution structural MRI (T1); complementing with iEEG and fMRI datasets that are shared in accompanying data papers. The data was obtained through an adversarial collaboration between advocates of two neuroscientific theories of consciousness: the Global Neuronal Workspace Theory and the Integrated Information Theory. The dataset includes recordings from 100 individuals (mean age 22.79 ± 3.59 years, 54 female, all right-handed) across two research centers (UK and China), using a standardized data collection protocol. During the experiment, participants were asked to perform a non-speeded Go/No-Go target detection task, during which they were exposed to visual stimuli from four distinct categories (faces, objects, letters, false fonts) presented at different orientations (front, left, right view), and for varying durations (0.5, 1.0, 1.5 s), under different task conditions. The quality of the data was assessed and organized according to the Brain Imaging Data Structure (BIDS). It is accompanied by extensive metadata to enhance reusability.

## Background & Summary

Understanding the relationship between consciousness and neural processes in the human brain is one of the most significant contemporary scientific challenges^1^. In a recent attempt to meet this challenge, the Cogitate adversarial collaboration^2,3^ contrasted two neuroscientific theories of consciousness: the Global Neuronal Workspace Theory (GNWT)^4,5^ and the Integrated Information Theory (IIT)^6,7^. With the proponents of the two theories, the Cogitate team has devised two experiments to test their respective predictions, embracing a rigorous experimental approach and adhering to the principles of open science^2,3,8^. Namely, we used large sample sizes to ensure sufficient power, collected at independent laboratories using distinct participant pools. This multimodal project produced three distinct data records acquired using three different neural techniques: functional magnetic resonance imaging (fMRI)^9^, intracranial electroencephalography (iEEG)^10,11^, and simultaneous magnetoencephalography (MEG) and electroencephalography (EEG)^12^. While employing an identical experimental paradigm to facilitate cross-modal comparison, these datasets were acquired from independent cohorts of participants (i.e., the participants in the MEG-EEG study did not participate in the fMRI or iEEG components). The present Data Descriptor focuses exclusively on the latter simultaneous MEG-EEG dataset. This comprehensive approach was taken to enhance sensitivity, spatiotemporal resolution, and whole-brain coverage to evaluate the hypotheses proposed by the theories. Now, these datasets could be used to explore additional questions, as suggested below.

The commitment to open science covered the full research cycle from idea to inception, whereby all experimental details, including the study rationale and predictions from the theories were preregistered (https://osf.io/92tbg/), and openly peer-reviewed^3^, to implementation and data release. The open research cycle is concluded with sharing all experimental code, analysis code as well as the behavioural, eye tracking and brain data paired with extensive metadata descriptors to facilitate reproducibility and more importantly reuse of this unique multimodal dataset.

In comparison with the Cogitate fMRI and iEEG datasets, the current MEG-EEG record offers distinct and complementary advantages. First, as opposed to iEEG, which is invasive and spatially restricted to clinical electrode placement, MEG-EEG is a non-invasive method that enables comprehensive whole-brain neural activity coverage and individual subject-based analysis. Second, as in contrast to fMRI, which is limited by the slow hemodynamic response, MEG-EEG provides superior millisecond-level temporal resolution while maintaining modest spatial resolution. This makes it particularly well-suited for exploring rapid spatiotemporal patterns and investigating the neural mechanisms and network interactions across diverse brain regions.

Currently, high-quality MEG-EEG datasets with a substantial sample size that specifically target the experience of visual consciousness are scarce. While initiatives such as the Human Connectome Project (HCP)^13^, The Cambridge Centre for Ageing and Neuroscience (Cam-CAN)^14^ and Welsh Advanced Neuroimaging Database (WAND)^15^ have advanced research on brain connectivity and age-related neural mechanisms, they differ significantly from the Cogitate dataset. Mainly, they were not collected in an attempt to study conscious perception: HCP focuses on macroscopic brain networks and individual differences between participants, and Cam-CAN and WAND emphasizes multimodal imaging across the lifespan.

In contrast, the Cogitate dataset is especially suitable for studying the mechanisms of perception and its relations with task performance. Thus, this dataset serves as a bridge connecting large-scale brain networks to investigations of fine-grained cognitive mechanisms. The Cogitate data has thus far been used to test core predictions derived from the adversarial collaboration^8^ pursuing a sophisticated falsification approach^16–18^. This project was designed to adjudicate between prominent theories of consciousness, specifically GNWT and IIT, by examining the anatomical localization and temporal dynamics of neural activity. Specifically, the experimental design and subsequent analyses focused on: (1) the anatomical localization of the neural correlates of consciousness (prefrontal vs. posterior cortex); (2) the timing of neural activity during sustained perception (transient vs. sustained responses); and (3) the patterns of functional connectivity, investigating how prefrontal executive areas and posterior sensory regions collaborate or synchronize during visual processing. These investigations utilized varied methodological approaches, such as temporal generalization^19^ and generalized eigendecomposition^20^. These findings demonstrate the high technical quality and internal consistency of the data in the context of pre-registered hypothesis testing.

Beyond its initial application, the dataset offers broad potential for the wider neuroscience community. Its high-density, synchronized EEG-MEG recordings are particularly suited for investigating fundamental mechanisms of visual perception and working memory, such as the maintenance of neural representations over time. Furthermore, the dataset can serve as a bridge connecting large-scale brain networks to fine-grained cognitive mechanisms. For instance, the multi-center BIDS structure provides the statistical power to model robust connectivity metrics, while the sub-millisecond temporal resolution allows for the development of advanced multimodal fusion techniques^21^ and cross-modal modeling with the consortium’s fMRI and iEEG cohorts. Accordingly, we hope that sharing the data with extensive metadata will facilitate data reuse and further exploration of questions in cognitive neuroscience as well as method development

Here, we accordingly share the data collected as part of the first experiment conducted in Cogitate^8^. The shared dataset includes concurrent MEG, EEG, and eye-tracking recordings from 100 participants recruited from two research centers in the United Kingdom and in China, following a standardized data collection protocol. In addition to the primary experimental data, we also collected empty-room and resting-state data, along with T1 structural scans for each participant, to support MEG source modelling.

## Methods

### Ethics and data privacy

This study was conducted in accordance with the Declaration of Helsinki and was approved by the institutional review boards (IRB) of the participating data collection sites. Specifically, the data collection at the University of Birmingham (UoB; Site A) was approved by the Science, Technology, Engineering and Mathematics Ethical Review Committee at the Centre for Human Brain Research, University of Birmingham (ERN_18-0226AP20). The data collection at Peking University (PKU; Site B) was approved by the committee for Protecting Human and Animal Subjects at school of psychological and cognitive sciences, Peking University (2020-05-07e).

Participants were recruited through campus-wide advertisements, including digital portals and physical posters at the respective universities. Prior to the experimental sessions, all participants provided written informed consent. This consent explicitly covered their participation in the MEG/EEG/MRI experiments, the storage of their data, and the public sharing and reuse of their de-identified datasets for research purposes within the scientific community.

To ensure the protection of participant privacy, a rigorous de-identification pipeline was applied to all data modalities prior to sharing. High-resolution T1-weighted MRI images were processed using a defacing algorithm to remove identifiable facial features. For MEG and EEG data, all personal identifying information (PII)—including names, exact dates of birth, and local hospital/lab IDs—was permanently stripped from the headers of both the originally formatted files and the BIDS versions (Brain Imaging Data Structure)^22^, which follows the specific extensions for MEG^23^. Similarly, eye-movement and behavioural data were thoroughly scrubbed to remove any sensitive metadata or local identifiers. Consequently, all shared data records across all modalities are fully de-identified, ensuring that participants cannot be re-identified while preserving the integrity of the scientific data for public reuse.

### Participants

**Fig. 1:**
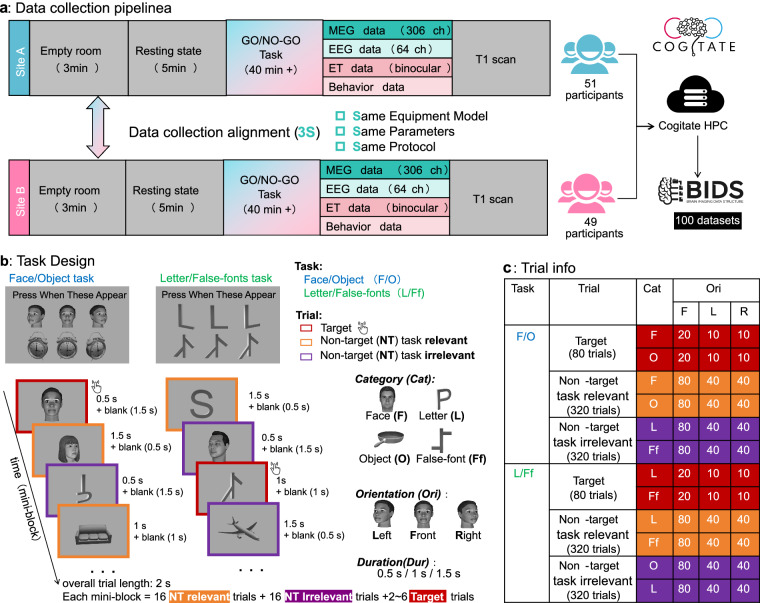
Data collection pipeline and experimental design. (**a**) The data acquisition was carried out at UoB and PKU (Site A and B) using standardized and comparable equipment, parameters, and protocols. (**b**) In the non-speeded Go/No-Go target detection task, for each mini-block, participants were required to detect two target stimuli belonging to two different categories (faces and objects, F/O, or letters and false fonts, L/Ff) presented at three different orientations (front, left, right views). They were instructed to press a button when one of the targets appeared within a sequence (34–38 trials) of visual stimuli. Since the response should have been independent of stimulus orientation, the latter was always task-irrelevant. The stimuli in the sequence were presented for varying durations (0.5 s, 1.0 s and 1.5 s), followed by blank intervals (500–1500 ms, overall trial length was 2 s). Task conditions included Target, Non-target Task Relevant (i.e., same category as the target), and Non-target Task Irrelevant trials (i.e., different category from the target), categorized by stimulus type (Face, Object, Letter, False font) and orientation (Left, Front, Right). (**c**) Detailed trial distributions are provided for each stimulus category (F/O and L/Ff), specifying trial counts per condition (e.g., for the F/O task, there were 80 target trials - 40 Face trials and 40 Object trials, each with 20 front, 10 left, and 10 right orientations).

One hundred and two healthy participants aged 18 years or older were enrolled in the project after providing informed consent, including the sharing and reuse of data. Following quality control procedures (detailed in the Technical Validation section), data from 100 participants were retained for the final release (mean age 22.8 ± 3.6 years, 54 female, all right-handed; with reportedly normal or corrected-to-normal visual acuity, and no known history of psychiatric or neurological disorders). The shared datasets (Fig. 1a) comprise behavioural, eye-tracking, MEG, and EEG data collected at the Centre for Human Brain Health at the University of Birmingham (UoB: 51 participants), and the Center for MRI Research at Peking University (PKU: 49 participants).

### Experimental design

Participants performed a non-speeded Go/No-Go target detection task with five critical experimental manipulations implemented in a factorial design (Fig. 1b). Stimuli were of (1) four different categories (faces, objects, letters and false fonts), (2) twenty identities per category, and were displayed (3) at three different orientations (front, left and right views) (4) for three different durations (0.5, 1.0 and 1.5 s), across (5) three task-relevance conditions (target, task-relevant, task-irrelevant). *Targets* were stimuli that participants had to detect among a series of serially-presented stimuli by pressing a button whenever one of these stimuli matched the target (e.g., the specific face or object defined at the beginning of the trial). *Task-relevant non-targets* were stimuli of the same category as the targets but of a different identity (e.g., a different face or object), and *Task-irrelevant non-targets* were stimuli of a different category than the targets (e.g., a letter or false font when looking for a face or object). In each mini-block, the participants’ task was to detect two targets: either a specific Face and a specific Object (F/O task), or a specific Letter and a specific False Font (L/Ff task). Each mini-block contained 16 non-target task relevant trials, 16 non-target task irrelevant trials, and 2 to 6 target trials (evenly distributed across mini-blocks; see Fig. 1b for trial sequence). At the beginning of every mini-block, the two target stimuli were switched to a new pair of images that did not repeat across any other mini-blocks throughout the block, ensuring that participants had to update their target templates frequently. The experiment followed a hierarchical structure: every 4 mini-blocks (two F/O and two L/Ff tasks, with order counterbalanced) were defined as a Block, after which participants could take a break, and every 2 blocks constituted a Run (after which MEG-EEG data was saved). Overall, the task included 10 blocks (i.e., 5 runs), resulting in 40 mini-blocks and a total of 1,440 trials. This design was strictly counterbalanced across the session for the four stimulus categories, 20 unique identities per category, and three orientations. The exact trial counts for each condition, which vary by design (e.g., target vs. non-target frequency), are detailed in the design metrics in Fig. 1c.

### Stimuli

The stimuli were presented foveally and occupied an area of approximately 6 by 6 degrees of visual angle on the screen. Faces were generated using FaceGen Modeler (Version 3.1; Singular Inversions, Toronto, Canada). Variation in hairstyles, ethnicities, and genders was incorporated into the faces to facilitate target individuation. Letters and false fonts stimuli were produced using Maxon Cinema 4D Studio (Version 20.059, Maxon Computer GmbH, Friedrichsdorf, Germany). Object stimuli were obtained from the Object Databank^24^. All stimuli were converted to grayscale and adjusted for uniform luminance and size. Stimulus orientation was evenly distributed, with half of each category presented in a side view (facing either left at 30° or right at −30°) and the remaining half in front view.

### Experimental procedure

For each participant, we first recorded 3 mins of the empty room signal in the MEG-shielded room (without the participant being present) on the day of their visit as a baseline reference. Prior to their participation in the non-speeded Go/No-Go target detection task, we collected 5 mins of eyes-open resting-state data. Participants were tasked with focusing on a white cross positioned on a gray background, maintaining stillness and blinking naturally. To localize the sources of the MEG data using personalized realistic head modelling, we obtained a high-resolution T1-weighted (T1w) MRI volume (3 T MAGNETOM Prisma scanner; Siemens Healthineers, Erlangen, Germany) for each participant.

### Data collection harmonization

To mitigate inconsistencies in data collection between the two sites, experimental devices and materials were standardized to the maximum extent possible. These standardized devices included: 1) a 306-channel whole-head TRIUX MEG system (MEGIN Oy, Helsinki, Finland) equipped with an integrated EEG system and a custom-made 64-channel electrode cap; 2) an MEG-compatible EyeLink 1000 Plus eye-tracker (SR Research Ltd., Ottawa, Canada); and 3) a PROPixx DLP LED visual stimulus projector (VPixx Technologies Inc., Saint-Bruno, Canada).

To make sure the experimental procedure was identical between the sites, a Standardized Operating Procedure (SOP) was developed, including precise instructions for the execution of the experiment^25^. The SOP encompassed participant protocols, setup guidelines, and overall experimental procedures. While ensuring uniformity in fundamental methodologies, the SOPs were tailored to address the distinct needs of the two site configurations. For full transparency, the detailed SOP documents—covering participant protocols and hardware setup—are hosted on the Cogitate Data Release Wiki (https://cogitate-consortium.github.io/cogitate-data/) under the ‘Links and Reference Materials’ section.

Additionally, we implemented a standardized framework to benchmark the experimental setup at the two sites, which was developed within the Cogitate consortium^26^. This framework includes quality checks for controlled event features, event timing, log file content validation, and peripheral trigger reliability, ensuring consistent experimental performance across sites, and mitigating malfunctions.

Moreover, to ensure data quality, we applied a systematic, multi-level quality checks protocol. This protocol focuses on post data collection checks to ensure data consistency, completeness and anonymization, ascertain participant compliance with the task, and verify the quality of the neural data (see Technical Validation section below), facilitating reliable hypothesis testing and data reuse.

### MEG-EEG Data Acquisition

The MEG systems consist of 204 planar gradiometers and 102 magnetometers, utilizing highly sensitive SQUID (Superconducting Quantum Interference Device) sensors arranged in a helmet-shaped array (Triux, MEGIN Oy, Helsinki, Finland). Simultaneous EEG data was recorded using an integrated EEG system and a 64-channel MEG-compatible EEG cap (MEGIN Oy, Helsinki, Finland). This cap was specifically designed by the manufacturer to be used in conjunction with the MEG system for simultaneous MEG-EEG recordings. The MEG systems are furnished with a zero boil-off Helium recycling system. They are housed in shielded rooms comprising two layers of mu-metal and one layer of aluminum as well as an active shielding system for further environmental noise reduction.

To achieve consistent coverage of frontal and posterior brain regions, the MEG gantry was set at a 68-degree angle. The MEG-EEG signals were sampled at 1000 Hz and underwent band-pass filtering between 0.01 and 330 Hz prior to sampling.

Bipolar electrodes were positioned on the participant’s chest (upper left and upper right) to capture the electrocardiogram (ECG). Additionally, two sets of bipolar electrodes were placed around the eyes to record the electrooculogram (vertical and horizontal EOG). EEG, ECG and EOG ground and reference electrodes were situated on the back of the neck and right cheek, respectively. The participant’s head position within the MEG system was monitored using four head position indicator (HPI) coils positioned on the EEG cap near the left and right mastoids and over the left and right frontal areas. The head fiducials’ positions, head shape, HPI position, and the position of the 64 EEG electrodes were recorded using a 3-D digitizer system (Polhemus Isotrak).

To ensure high signal integrity of EEG signal, electrode impedances were checked and maintained below 10 kΩ for all 64 channels. During the acquisition, since the simultaneous MEG-EEG setup does not allow for real-time impedance monitoring, trained technicians continuously performed online visual inspection of the raw EEG traces. Any sensors exhibiting persistent artifacts or drift were meticulously documented in a Participants Case Report Form (CRF). These real-time quality logs are preserved and shared as *CRF.json files for each participant, providing a transparent record of the raw data collection conditions.

### Anatomical MRI data acquisition

High-resolution T1-weighted MRI volumes to be used for source modelling were obtained for each participant using a 3 T Siemens MRI Prisma scanner. At the UoB, a 32-channel coil captured images with a resolution of 1 × 1 × 1 mm, 208 sagittal slices, and a field of view (FOV) of 256 × 256 matrix. At PKU, a 64-channel coil with a resolution of 0.5 × 0.5 × 1 mm, 192 sagittal slices, and a FOV of 224 × 256 matrix was employed. To mitigate potential interference from body magnetization during MEG recordings, all MRI scans were conducted at least one week prior to or following the MEG session. A detailed list of participants with missing MRI data is provided in the Technical Validation section.

### Behavioural setup

Visual stimuli were displayed on a screen positioned in front of the participants using a PROPixx DLP LED projector (VPixx Technologies Inc, Saint-Bruno, Canada) operating at a resolution of 1920 × 1080 pixels and a refresh rate of 120 Hz. The distance between the participants’ eyes and the screen varied across sites (UoB: 119 cm, PKU: 85 cm) to account for disparities in optical path design and resultant screen size variations, ensuring a consistent field of view measuring 36.6 × 21.2 degrees. Participants provided manual responses using two 5-button response boxes. Although the specific hardware models differed between sites (UoB: NAtA, NAtA Technologies, Kotka, Finland; PKU: SINORAD, Shenzhen Sinorad Medical Electronics Co., Ltd, Shenzhen, China), the button mapping and response contingencies were standardized across data collection sites.

### Eye tracking

Eye movements were monitored and recorded using a MEG-compatible EyeLink 1000 Plus eye-tracker (SR Research Ltd., Ottawa, Canada) in a binocular manner. Calibration with nine points was conducted initially and recalibrated at the start of each block if needed. Pupil size and corneal reflection data were sampled at a rate of 1000 Hz.

## Data Records

The dataset is permanently archived in the Cogitate data repository (hosted by the Max Planck Society) and can be accessed via two persistent identifiers: 10.17617/1.WQA3-WK71^27^ for the originally formatted data (shared under the record name ‘Cogitate M-EEG Data’ on the data repository) and 10.17617/1.nzwp-7j89^12^ for the BIDS data (shared under the record name ‘Cogitate M-EEG BIDS Data’ on the data repository). Within each DOI repository, the records are organized as versioned, bundled archives to ensure long-term stability and reproducibility, independent of the project’s interactive web-interface. To accommodate different research needs, we provide two download options: a Sample Data bundle (~28 GB), which includes four representative participants for rapid local testing of analysis pipelines, and a Full Data bundle (~700 GB), containing the complete multi-center dataset. In addition to the static bundled archives, granular browsing of the dataset is supported to facilitate data discovery (see Usage Notes for details on interactive access).

Adhering to the principles of Open Science and the FAIR (Findable, Accessible, Interoperable, and Reusable) data standards, we provide the data in these two parallel forms to ensure maximum transparency and provenance. While the BIDS version facilitates standardization and ease of use, the originally formatted data is retained to provide the complete, native acquisition structure (excluding personal identifiers). This ensures that all proprietary metadata and auxiliary files remain accessible in their unaltered state, allowing researchers to reference the exact data landscape prior to BIDS conversion. Conversely, the BIDS version is optimized for immediate reuse with standardized analysis pipelines, ensuring that the community can verify every step of our data standardization while maintaining long-term data integrity.

The naming conventions for originally formatted data and BIDS data described below are consistent across all shared data records. For anatomical MRI, data are provided in both their originally formatted (DICOM) and BIDS (NIFTI) versions to ensure maximum flexibility and data provenance. This dual-format approach preserves the full suite of metadata fields contained within the original DICOM headers, some of which may be omitted or altered during the standardized transformation to the NIfTI format.

### Originally formatted data structure

**Fig. 2:**
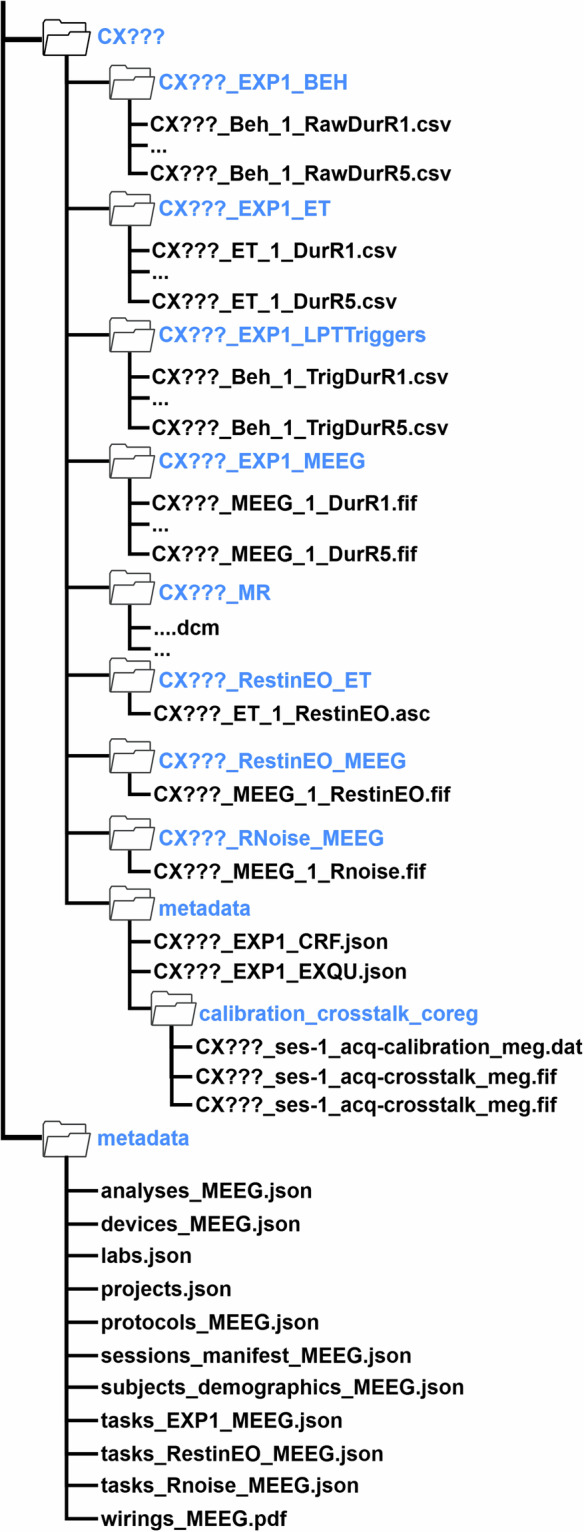
Structure of originally formatted data. The details for each folder and file see Tables 1, 2.

The originally formatted data structure consists of participant-specific folders, along with a project metadata folder (see Fig. 2). The participant’s folder is labeled using the format *CX???*, where the first two letters reflect the site where the data were collected (*CA*: UoB; *CB*: PKU), and the question marks represent the participant ID (e.g., *CA101*). Details for participant-specific folders are summarized in Table 1, while details for the project metadata folder are summarized in Table 2.

**Table 1:** Details for participant-specific folders (/CX???).

Level	Folder Name	Folder Description	File Description	Data format	Data type	Example Filename
1	CX???	participant-specific folder	/	/subfolder	/	CA101/
2	CX???_EXP1_BEH	behavioural data for experiment	behavioural log files for each run	csv	behavioural data	CA101_Beh_1_RawDurR3.csv
2	CX???_EXP1_ET	eye tracking data for experiment	eye tracking data for each run	ascii	eye tracking data	CA101_ET_1_DurR2.asc
2	CX???_EXP1_LPTTriggers	LPT trigger data for experiment	parallel port triggers used to mark events in the MEG-EEG data	csv	LPT trigger	CA101_Beh_1_TrigDurR4.csv
2	CX???_EXP1_MEEG	MEG-EEG data for experiment	MEG-EEG data for each run of the experiment	fif	MEG-EEG data	CA101_MEEG_1_DurR5.csv
2	CX???_MR	participant-specific anatomical MRI scan	participant-specific anatomical MRI scan	dcm	anatomical MRI scan	CA109_MR_101.dcm
2	CX???_RestinEO_ET	eye tracking data for eye-open resting state	eye tracking data for eye-open resting state	ascii	eye tracking data	CA101_ET_1_RestinEO.asc
2	CX???_RestinEO_MEEG	MEG-EEG data for eye-open resting state	MEG-EEG data for eye-open resting state	fif	MEG-EEG data	CA101_MEEG_1_RestinEO.fif
2	CX???_RNoise_MEEG	MEG-EEG data for empty room	MEG-EEG data for empty room	fif	MEG-EEG data	CA101_MEEG_1_Rnoise.fif
2	metadata	participant-specific metadata	document listing any issues encountered during data collection	json	Case Report Form	CA101_EXP1_CRF.json
			post-task debriefing questionnaire completed by participants after the experiment to provide feedback on their experience with the tasks performed	json	Exit Questionnaire	CA101_EXP1_EXQU.json
3	calibration_crosstalk_coreg	transformation matrix for the MRI-MEG coregistration	MEG crosstalk and fine-calibration files required for SSS/maxfilter during MEG data preprocessing	dat/fif	MEG fine-calibration files/crosstalk file	CA109_ses-1_acq-crosstalk_meg.dat/CA109_ses-1_acq-calibration_meg.fif

**Table 2:** Details for the project metadata folders (/metadata).

Filename	File Description	Data formats	Data type
analysis_MEEG.json	the link to the analysis scripts used in Cogitate	json	analysis scripts link
devices_MEEG.json	a list of data acquisition devices	json	data acquisition devices list
labs.json	metadata related to the project partner’s laboratories, such as institutional name, location, contact details, and Principal Investigator information	json	labs info
projects.json	project-related metadata, including start and end dates, funding details, project personnel, outputs, and ethical considerations	json	projects info
protocols_MEEG.json	the experimental protocol	json	the experimental protocol
sessions_manifest_MEEG.json	the MR dataset manifest	json	MR manifest
subjects_demographics_MEEG.json	participant demographic data	json	participant demographic
Tasks_EXP1_MEEG.json	descriptions of the experimental MEG-EEG data collection procedure	json	experiment procedure
Tasks_RestinEO_MEEG.json	descriptions of the resting state with open eye MEG-EEG data collection procedure	json	resting state procedure
Tasks_Rnois_MEEG.json	descriptions of the empty room MEG-EEG data collection procedure	json	empty room procedure
wirings_MEEG.pdf	a wiring diagram for device connections	pdf	wiring diagram for MEG device

### BIDS data structure

**Fig. 3:**
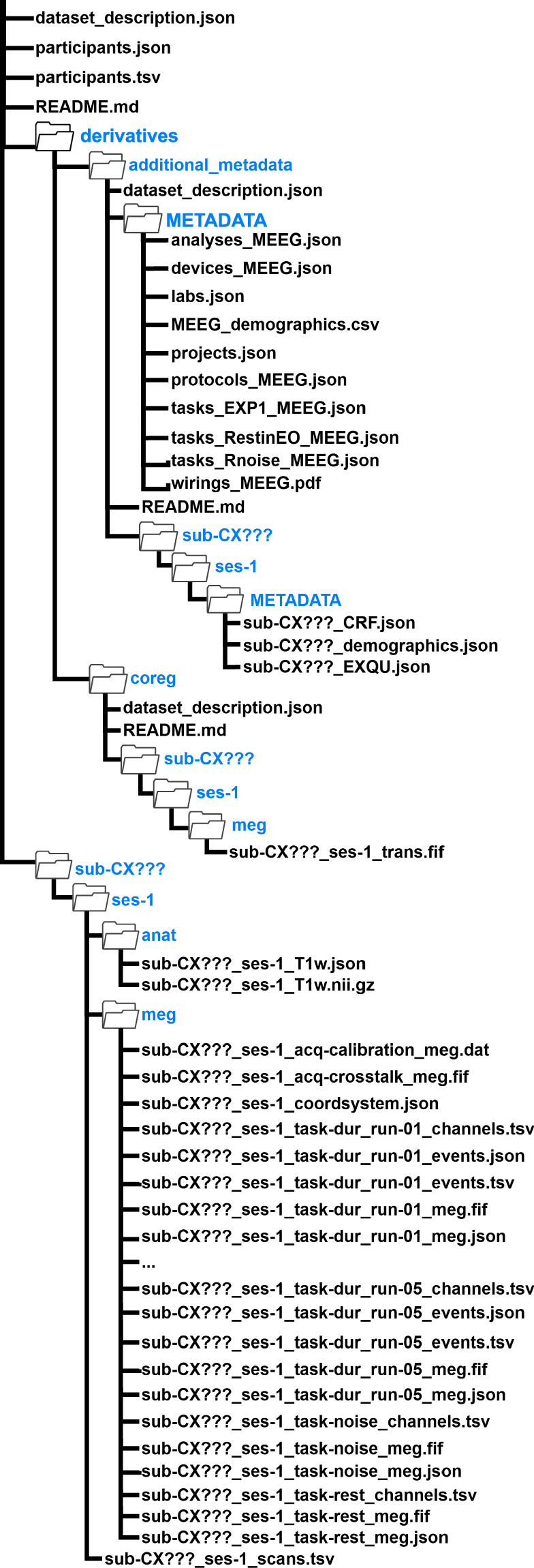
Structure of BIDS data. The BIDS root directory contains a participant-specific folder (*sub-CX???*), which includes a session folder (*ses-1*) housing the MEG-EEG (*meg*) and MRI (*anat*) data folders. The MEG-EEG folder (*meg*) contains the MEG-EEG data (*sub-CX???_ses-1_task-TASKNAME[_run-??]_meg.fif*; the *run* field is omitted in the case of resting [*rest*] and empty-room [*noise*] data*)*, sidecar JSON file details about the MEG data acquisition (*sub-CX???_ses-1_task-TASKNAME[_run-??]_meg.json*), coordinates of anatomical landmarks and head-localisation coils (*sub-CX???_ses-1_coordsystem.json)*, events associated with the experimental task (*sub-CX???_ses-1_task-TASKNAME_events.tsv)*, JSON file containing information about the events (*sub-CX???_ses-1_task-TASKNAME_events.json)*, channel information (*sub-CX???_ses-1_task-Dur_channels.tsv*), fine-calibration and crosstalk files (*sub-CX???_ses-1_acq-calibration_meg.dat* and *sub-CX???_ses-1_acq-crosstalk_meg.fif)*. MRI folder (*anat*) contains the anatomical MRI scan (*sub-CX???_ses-1_T1w.nii.gz*) and a sidecar JSON file with details about the MRI scan acquisition. The *derivatives/coreg* folder contains the transformation matrix for the MRI-MEG coregistration (*sub-CX???_ses-1_trans.fif)*. Additional metadata (case report forms, exit questionnaires, subject demographics, information related to the experimental setup, etc.) are included along with the BIDS data under *derivatives/additional_metadata*.

The BIDS-converted data are organized according to the BIDS convention for MEG^23^ and converted using the MNE-BIDS toolbox^28^ (see Fig. 3). The dataset’s metadata is organized to support evaluation at both the cohort and individual levels. Summary metadata for the collection as a whole, including participants.tsv and dataset_description.json, are located in the root directory to allow for a rapid assessment of the repository’s overall content and demographic composition. Individual-level metadata are provided via participant-specific JSON sidecars (e.g., *_MEEG.json, *_T1w.json, and task-related.json files) within each participant’s folder. These sidecars provide detailed acquisition parameters for each specific participant, ensuring transparency and facilitating data reuse. Details of naming conventions and formats of BIDS data can be found in Table 3.

**Table 3:** Naming conventions and formats of the BIDS data.

Data type	File naming conventions	Data formats
MEG-EEG data (experimental task, empty room, and resting-state)	sub-CX???_ses-1_task-*TASKNAME*[_run-??]_meg	FIF
MEG-EEG sidecar information	sub-CX???_ses-1_task-*TASKNAME*_meg	JSON
Channels information	sub-CX???_ses-1_task-*TASKNAME*_channels	TSV
Experimental events	sub-CX???_ses-1_task-*TASKNAME*_events	TSV
Events description	sub-CX???_ses-1_task-*TASKNAME*_events	JSON
Coordinates of anatomical landmarks and head-localization coils	sub-CX???_ses-1_coordsystem	JSON
Fine-calibration file (for SSS/maxfilter)	sub-CX???_ses-1_acq-calibration_meg	DAT
Crosstalk file (for SSS/maxfilter)	sub-CX???_ses-1_acq-crosstalk_meg	FIF
MR scan	sub-CX???_ses-1_T1w.nii.gz	NIFTI

Each participant’s folder comprises nine data types: MEG-EEG data, MEG-EEG sidecar information, channels information, experimental events, events, description, coordinates of the anatomical landmarks and head-localization coils, fine-calibration and crosstalk files, and MR scan. All data folders and files adhere to the BIDS naming convention *sub-CX???_ses-1_task-TASKNAME[_run-??]_DATATYPE*. The specific data format for each data type is detailed in the table.

To facilitate source space analysis, the BIDS collection includes anatomical landmarks (NAS, LPA, and RPA fiducials) documented in the BIDS data. These landmarks are provided in the head coordinate system (MNE/Neuromag conventions). Crucially, for each participant with an available MRI, we provide a pre-calculated MEG-to-MRI transformation matrix (*_trans.fif file). This file ensures accurate co-registration between the MEG sensors and the structural MRI, accounting for the defacing of the anatomical images. While these files are natively generated in MNE-Python^29^, they are compatible with other major analysis toolboxes, such as FieldTrip^30^, SPM^31^, and Brainstorm^32^ ensuring that the dataset is accessible for various source reconstruction pipelines.

The BIDS root directory contains the participant’s folders, also labelled using the convention of *sub-CX???*. Each participant’s folder contains a nested folder structure, with the highest level referring to the session of the recording (*ses-1*), followed by the MEG-EEG data folder (*meg*) and anatomical MR data folder (*anat*). All data files are located at the lowest level of the directory structure (see Fig. 3). The MEG-EEG data are stored in the FIF format following the naming convention *sub-CX???_ses-1_task-TASKNAME[_run-??]_meg*. The sidecar JSON file with the same name contains details about the MEG data acquisition (e.g., sampling rate, number of channels, filtering settings). The coordinates of anatomical landmarks and HPI coils are located in a JSON file named *sub-CX???_ses-1_coordsystem*. The events associated with the experimental task (i.e., MEG-EEG triggers) are stored in a TSV file under the name *sub-CX???_ses-1_task-TASKNAME_events* and are accompanied by a JSON file of the same name containing information about the events. In addition, the *sub-CX???_ses-1_task-Dur_channels* TSV file contains additional information about each channel in the recording, such as the type of sensor (magnetometer, gradiometer, EEG, EOG, ECG, trigger and MISC), online filtering used during data collection, and indications about the channel status (good or bad). The fine-calibration and crosstalk files required to apply Maxwell filtering/SSS (Signal Space Separation) using the open-source MNE-python^29^ package are stored under the names *sub-CX???_ses-1_acq-calibration_meg.dat* and *sub-CX???_ses-1_acq-crosstalk_meg.fif*, respectively. Additional metadata (case report forms, exit questionnaires, participant demographics, information related to the experimental setup, etc.) are included along with the BIDS data under *derivatives/additional_metadata*. A breakdown of the file types and formats can be found in Table 2.

### Participant and experimental metadata

Metadata for individual participants and at the experiment level are available through custom forms within the XNAT graphical user interface. The “Resources” tab contains participant-level metadata and can be accessed and downloaded on each participant’s page. Case Report Forms (CRFs, documents listing any issues encountered during data collection) and experiment questionnaires (EXQU, post-task debriefing questionnaire completed by participants after the experiment to provide feedback on their experience with the tasks performed) are stored per session and can thus be accessed and downloaded from the “Resources” tab of each experimental session.

## Technical Validation

**Fig. 4:**
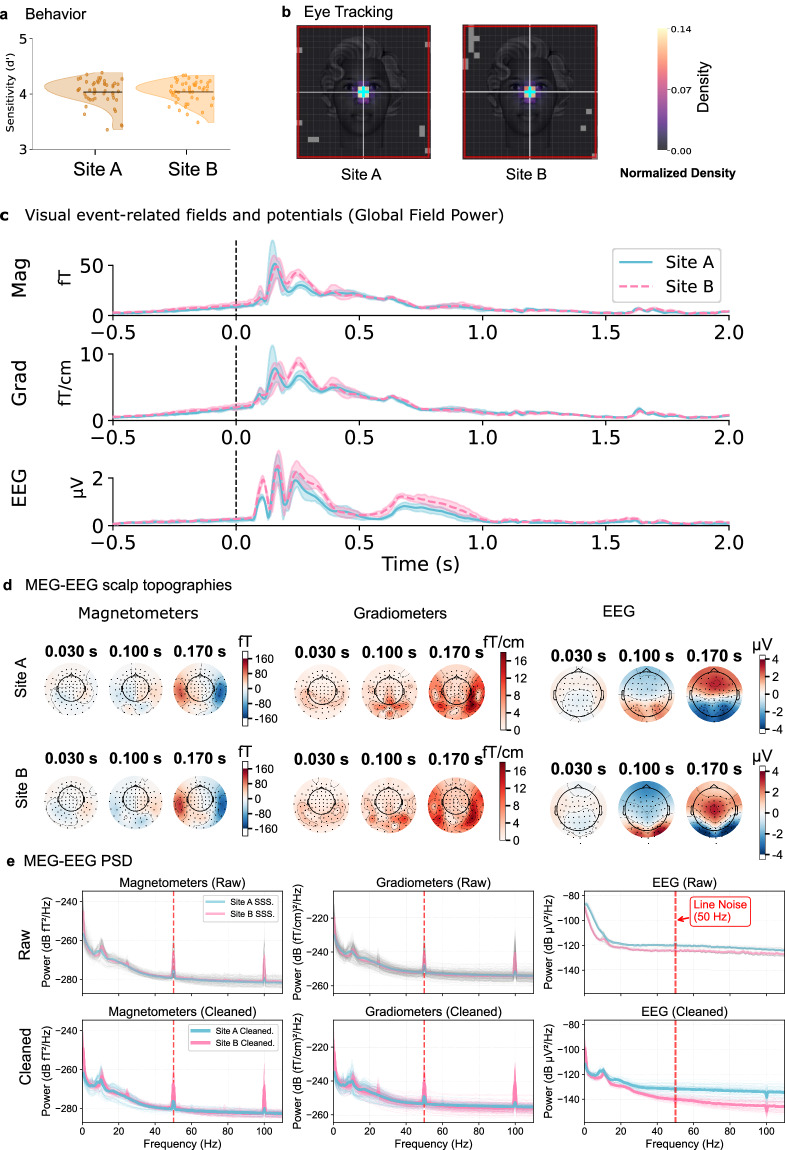
Technical Validation data. (**a**) The distribution of behavioral sensitivity scores (d’) is shown separately for each data acquisition site. Horizontal black lines indicate the average d’ for each site, while individual participant d’ scores are represented by dots. (**b**) Zoomed-in fixation density maps are shown separately for each data acquisition site. They were generated over a 0.5 s interval following stimulus onset. The display area is restricted to the central stimulus presentation region (indicated by the red boundary box) to visualize fixation stability. The cyan crosshair marks the central fixation point. Note the high density of gaze points clustered tightly around the fixation cross across sites. (**c**) **Visual event-related fields and potentials:** Global Field Power (GFP) of grand-averaged visual evoked responses for the MEG (gradiometers (grad) and magnetometers (mag)) and EEG data. Solid lines represent the mean response and shaded areas the standard deviation, illustrating high temporal alignment between Site A (cyan) and Site B (pink). (**d**) **Scalp Topographies:** Topographical displays of the grand average signal at 30 ms, 100 ms and 170 ms. These plots are shown for each data acquisition site, underscoring the consistency across sites. (**e**) **Power Spectral Density (PSD) validation comparing representative participants**. The top row displays Maxwell filtering SSS (MEG) and raw (EEG) data with characteristic 50 Hz line noise. The bottom row shows the cleaned data (consistent with panels c-d), demonstrating effective noise suppression and preserved physiological signals (e.g., alpha peaks at ~10 Hz). Faint traces indicate individual channels; thick lines represent the median power. Data in **c-e** underwent minimal preprocessing to demonstrate signal quality, including Maxwell filtering (MEG), bad channel detection via PREP (EEG), 0.1–40 Hz band-pass filtering, ICA-based artifact removal, and epoch rejection. All data analysis and visualization were performed using MNE-Python^29^; see the Code Availability section for specific software versions.

The data underwent a three-tier validation process. The first stage ensured that all necessary files were present, consistently named, and free of personal identifiers to maintain participant privacy. The second stage verified the integrity of the eye-tracking (Fig. 4a) and behavioral data (Fig. 4b). Task performance was analyzed to confirm that behavior was as expected, making sure participants followed instructions, and to ensure consistency across sites. The third stage consisted of multiple assessments to ensure the quality of the MEG-EEG data across sites. Each recording was inspected for overall noise level, alignment of sensor position to expected anatomical positions (one participant showed irreconcilable reconstruction of sensor positions), and presence of visual neural responses (Fig. 4c,d). Noise was inspected by comparing power spectra between empty-room recordings, resting-state sessions prior to the experiment, and experimental blocks.

From an original sample of 102 participants, a total of 100 datasets are included in the current release. Two participants (CA101 and CB082) did not meet the behavioral performance criteria (second level checks). Their hit rates were 49% and 78%, which was below the threshold of 80% required for inclusion in the primary Cogitate study analyses^3^. Although excluded from the main study, we decided to include these two datasets in the current database as they may remain valuable for other research purposes, such as investigating error-related neural activity. In contrast, two participant was further excluded during the third level quality checks due to excessive noise from the MEG sensors, which was estimated during preprocessing following the FLUX pipeline^25^. This was done with a global rejection threshold of peak-to peak amplitude, which was detected in more than 60% of the trials for this participant. Unlike the behavioral exclusions, these two datasets with severe quality issues were not released with this dataset, resulting in a final sample of 100 participants. Within this shared sample, some of the shared datasets are incomplete. Four participants (CA101, CA102, CA103, CA104) do not include EEG recordings, and six participants (CA101, CA102, CA104, CA110, CA111, CA152) lack individualized anatomical MRI data. Moreover, some eye-tracking (ET) files for individual blocks are missing for five participants (CA151, CA105, CA172, CA176, CB999).

Behaviorally, the participants included in the dataset exhibited a high mean hit rate (97.5%, SD = 2.4%) and a low mean false alarm rate (0.6%, SD = 0.6%). Eye-tracking data indicated stable fixation accuracy, with an average accuracy of fixation of 93.1% (SD = 7.4%) within 2 degrees of visual angle throughout the experiment (Fig. 4a-b).

To assess signal quality and cross-site consistency, MEEG data underwent a standardized preprocessing pipeline following the FLUX recommendations^8,25^. For MEG data, Maxwell filtering (Signal Space Separation, SSS) was applied to suppress external interference. For EEG data, noisy channels were automatically identified using the PREP pipeline^33^. Subsequently, both MEG and EEG data were band-pass filtered (0.1–40 Hz) to isolate task-relevant frequencies. Artifacts related to eye movements and cardiac activity were removed using Independent Component Analysis (ICA), and bad epochs were rejected based on absolute amplitude thresholds. As shown in Fig. 4, the resulting data demonstrate high data quality and comparability between Site A and Site B. The grand-average evoked responses (Fig. 4C) and scalp topographies (Fig. 4D) reveal consistent signal morphologies and spatiotemporal patterns across sites. Furthermore, the Power Spectral Density (PSD) analysis (Fig. 4E) confirms the expected 1/f spectral characteristics and comparable signal-to-noise profiles, validating the reliability of the dataset for cognitive neuroscience research.

## Usage Notes

The dataset is primarily archived as static bundled records (see Data Records) and is also accessible on an interactive XNAT platform (eXtensible Neuroimaging Archive Toolkit; http://cogitate-data.ae.mpg.de/), a system used by major projects like the Human Connectome Project (HCP)^13^.

This platform allows detailed exploration and selective retrieval without downloading the entire 700 GB repository. Researchers can navigate the complete BIDS directory structure and review metadata files online via the XNAT interface, without accessing the actual neuroimaging data. This feature enables immediate assessment of available parameters and records for each participant, enabling users to randomly select and assess individual datasets to confirm data integrity at the subject level before downloading. This granular approach meets the demand for adaptable, non-exclusive data auditing, offering accessibility and transparency beyond what static bundled archives offer alone.

Moreover, the platform supports the XNAT API (https://wiki.xnat.org/documentation/the-xnat-api) for automated and reproducible data retrieval, allowing researchers to programmatically filter and extract specific data subsets directly into their analysis workflows.

Detailed technical information, including comprehensive descriptions of experimental paradigms, data acquisition protocols, and BIDS conversion guidelines, can be found in our project Wiki (https://cogitate-consortium.github.io/cogitate-data/). This documentation serves as a central resource for the scientific community, ensuring the dataset’s long-term utility by providing essential context for its reuse and further analysis.

In the XNAT projects COG_MEEG_EXP1 and COG_MEEG_EXP1_SAMPLE, participants’ data is organized in the “Subjects” table, with each subject assigned a unique ID (“CX???”). Metadata is available under the “Details” tab (in “Custom Fields”) and in the “Resources” tab at the project level. Each subject’s directory includes an “Experiments” table with entries for “EyeTracker”, “MEG-EEG”, and “MR Session”. Subject-specific metadata (e.g., participant demographics) is provided as a custom field and as downloadable files in the “Resources” section. The “MEG-EEG” section contains individual recordings, while files like CRF, EXQU, crosstalk, calibration, and trans are stored in the “METADATA” folder under “Resources”, alongside behavioral and LPT trigger data.

For BIDS data, the XNAT structure mirrors the one of the originally formatted data, with participants listed in the “Subjects” table using the ID format “sub-CX???”. Metadata is accessible under the “Details” tab, following the same structure as originally formatted data bundles. Unlike originally formatted data, BIDS projects lack experimental entries for previewing recordings. Users can preview and download files via the “Resources” or “Manage Files” tabs, with filtering options available using the “Advanced” feature next to the search box.

## Data Availability

The datasets described in this Data Descriptor are available in the Cogitate data repository (hosted by the Max Planck Society). The originally formatted data can be accessed via 10.17617/1.WQA3-WK71, and the BIDS-formatted data can be accessed via 10.17617/1.nzwp-7j89.

## Code availability

The MATLAB code utilized for conducting the experimental task can be retrieved from https://github.com/Cogitate-consortium/cogitate-experiment-code/tree/MEEG-Exp1. The code for preprocessing pipeline and analysis scripts is accessible at https://github.com/Cogitate-consortium/cogitate-msp1/tree/main/coglib/meeg. All code is written in Python using MNE-Python^29^. The README file in the repository offers an overview of the codebase and detailed instructions for setting up the computational environment, including the specific software versions and dependencies required to reproduce the analysis. While the README provides a general guide, we refer users to the Cogitate Data Release Wiki (https://cogitate-consortium.github.io/cogitate-data/) for detailed usage instructions. This wiki documentation demonstrates how to download the data from our repository and execute the described analyses. It also hosts the Standardized Operating Procedures (SOPs) for data acquisition, along with further guidance on interacting with the dataset, such as selecting specific conditions and customization options.
